# A Db-Scan Binarization Algorithm Applied to Matrix Covering Problems

**DOI:** 10.1155/2019/3238574

**Published:** 2019-09-16

**Authors:** José García, Paola Moraga, Matias Valenzuela, Broderick Crawford, Ricardo Soto, Hernan Pinto, Alvaro Peña, Francisco Altimiras, Gino Astorga

**Affiliations:** ^1^Pontificia Universidad Católica de Valparíso, 2362807 Valparaíso, Chile; ^2^Universidad de Valparaíso, 2361864 Valparaíso, Chile

## Abstract

The integration of machine learning techniques and metaheuristic algorithms is an area of interest due to the great potential for applications. In particular, using these hybrid techniques to solve combinatorial optimization problems (COPs) to improve the quality of the solutions and convergence times is of great interest in operations research. In this article, the db-scan unsupervised learning technique is explored with the goal of using it in the binarization process of continuous swarm intelligence metaheuristic algorithms. The contribution of the db-scan operator to the binarization process is analyzed systematically through the design of random operators. Additionally, the behavior of this algorithm is studied and compared with other binarization methods based on clusters and transfer functions (TFs). To verify the results, the well-known set covering problem is addressed, and a real-world problem is solved. The results show that the integration of the db-scan technique produces consistently better results in terms of computation time and quality of the solutions when compared with TFs and random operators. Furthermore, when it is compared with other clustering techniques, we see that it achieves significantly improved convergence times.

## 1. Introduction

In recent years, different optimization methods based on evolutionary concepts have been explored. These methods have been used to solve complex problems, therein obtaining interesting levels of performance [1–3], and many such methods have been inspired by concepts extracted from abstractions of natural or social phenomena. These abstractions can be interpreted as search strategies according to an optimization perspective [4]. These algorithms are inspired, for example, by the collective behavior of birds, e.g., the cuckoo search algorithm [5]; the movement of fish, e.g., the artificial fish swarm algorithm (AFSA) [6]; particle movement, e.g., particle swarm optimization (PSO) [7]; the social interactions of bees (ABC) [8]; and the process of musical creation, as in the search for harmony (HS) [9] and in genetic algorithms (GA) [10], among others. Many of these algorithms work naturally in continuous spaces.

On the other hand, lines of research that allow robust algorithms associated with the solution of combinatorial optimization problems (COPs) to be obtained are of great interest in the areas of computer science and operations research. This interest is currently mainly related to decision making in complex systems. Many of these decisions require the evaluation of a very large combination of elements in addition to having to solve a COP to find a feasible and satisfactory result. Examples of COPs can be found in the areas of logistics, finance, transportation, biology, and many others. Depending on the definition of the problem, many COPs can be classified as NP-hard. Among the most successful ways to address such problems, one common method is to simplify the model to attempt to solve instances of small to medium size using exact techniques. Large problems are usually addressed by heuristic or metaheuristic algorithms.

The idea of hybridization between metaheuristic techniques and methods from other areas aims to obtain more robust algorithms in terms of solution quality and convergence times. State-of-the-art proposals for hybridization mainly include the following: (i) *mateheuristics*, which combines heuristics or metaheuristics with mathematical programming [11]; (ii) *hybrid heuristics*, which corresponds to the integration of different heuristic or metaheuristic methods [12]; (iii) *simheurístics*, which combines simulation and metaheuristics [13]; and (iv) the hybridization between metaheuristics and machine learning [14]. Machine learning can be considered as a set of algorithms that enable the identification of significant, potentially useful information and learning through the use of data. In this work, useful information will be obtained using the data generated by a continuous metaheuristic algorithm through the use of the db-scan unsupervised learning technique to obtain robust binarizations of this algorithm.

Within the lines of this discussion, we aim to provide the following contributions:

- A novel automatic learning binarization algorithm is proposed to allow metaheuristics commonly defined and used in continuous optimization to efficiently address COPs. This algorithm uses the db-scan unsupervised learning technique to perform the binarization process. The selected metaheuristics are particle swarm optimization (PSO) and cuckoo search (CS). Their selection is based on the fact that they are commonly used in continuous optimization and enable a method for adjusting their parameters in continuous spaces.
- These hybrid metaheuristics are applied to the well-known set covering problem (SCP). This problem has been studied extensively in the literature, and therefore, there known instances where we can clearly evaluate the contribution of the db-scan binarization operator. On the other hand, the SCP has numerous practical real-world applications such as vehicle routing, railways, airline crew scheduling, microbial communities, and pattern finding [15–18].
- Random operators are designed to study the contribution of the db-scan binarization algorithm in the binarization process. Additionally, the behavior of db-scan binarization is studied, comparing it with binarization methods that use *k*-means and transfer functions (TFs). Finally, the binarizations obtained by db-scan are used to solve a real-world problem.

The remainder of this article is structured as follows.  Section 2 describes the SCP and some of its applications. In Section 3, a state-of-the-art hybridization between the areas of machine learning and metaheuristics is provided, and the main binarization methods are described. Later, in Section 4, the proposed db-scan algorithm is detailed. The contributions of the db-scan operator are provided in Section 5. Additionally, in this section, the db-scan technique is studied by comparing it with other binarization techniques that use *k*-means and TFs as a binarization mechanism. In Section 6, a real-world application problem is solved. Finally, in Section 7, conclusions and some future lines of research are given.

## 2. Set Covering Problem

SCP is one of the oldest and most-studied optimization problems and is well known to be NP-hard [19]. Nevertheless, different solution algorithms have been developed. There exist exact algorithms that generally rely on the branch-and-bound and branch-and-cut methods to obtain optimal solutions [20, 21]. These methods, however, struggle to solve SCP instances that grow exponentially with the problem size. Even medium-sized problem instances often become intractable and can no longer be solved using exact algorithms. To overcome this issue, different heuristics have been proposed [22, 23].

For example, [22] presented a number of greedy algorithms based on a Lagrangian relaxation (called Lagrangian heuristics). Caprara et al. [24] introduced relaxation-based Lagrangian heuristics applied to the SCP. Metaheuristics, e.g., genetic algorithms [25], simulated annealing [26], and ant colony optimization [27], have also been applied to solve the SCP. More recently, swarm-based metaheuristics, such as the cat swarm [28], cuckoo search [29], artificial bee colony [8], and black hole [30] metaheuristics, have also been proposed.

The SCP has many practical applications in engineering, e.g., vehicle routing, railways, airline crew scheduling, microbial communities, and pattern finding [15, 16, 18, 31].

The SCP can be formally defined as follows. Let *A*=(*a*_*ij*_) be an *n* × *m* zero-one matrix, where a column *j* covers a row *i* if *a*_*ij*_=1, and a column *j* is associated with a nonnegative real cost *c*_*j*_. Let *I*={1,…, *n*} and *J*={1,…, *m*} be the row and column set of *A*, respectively. The SCP consists of searching a minimum cost subset *S* ⊂ *J* for which every row *i* ∈ *I* is covered by at least one column *j* ∈ *J*, i.e.,

(1) $$\begin{array}{c} \text{minimize }f\left(x\right)=\overset{m}{\underset{j=1}{\sum }}c_{j}x_{j}, \end{array}$$

(2) $$\begin{array}{c} \text{subject to}\overset{m}{\underset{j=1}{\sum }}a_{ij}x_{j}\geq 1,\;\forall i\in I,\text{ and }x_{j}\in \left\{0,1\right\},\;\forall j\in J, \end{array}$$

where *x*_*j*_=1 if *j* ∈ *S* and *x*_*j*_=0, otherwise.

## 3. Related Work

### 3.1. Related Binarization Work

A series of metaheuristic algorithms designed to work in continuous spaces have been developed. Particle swarm optimization (PSO) and cuckoo search (CS) are two of the most commonly used metaheuristic algorithms. On the other hand, the existence of a large number of *𝒩𝒫*-hard combinatorial problems motivates the investigation of robust mechanisms that allow these continuous algorithms to be adapted to discrete versions.

In a review of the state-of-the-art binarization techniques [32], two approximations were identified. The first approach considers general methods of binarization. In those general methods, there is a mechanism that allows the transformation of any continuous metaheuristic into a binary one without altering the metaheuristic operators. In this approach, the main frameworks used are TFs and angle modulation. The second approach corresponds to binarizations in which the method of operating metaheuristics is specifically altered. Under this second approach, notable techniques include quantum binary and set-based approaches.

#### 3.1.1. Transfer Functions

The simplest and most widely used binarization method corresponds to TFs. TFs were introduced by [33] to generate binary versions of PSO. This algorithm considers each solution as a particle. The particle has a position given by a solution in an iteration and a velocity that corresponds to the vector obtained from the difference of the particle position between two consecutive iterations. The TF is a very simple operator and relates the velocity of the particles in PSO with a transition probability. The TF takes values from *ℝ*^*n*^ and generates transition probability values in [0,1]^*n*^. The TFs force the particles to move in a binary space. Depending on the function's shape, they are usually classified as S-shape [34] and V-shape functions [1]. Once the function produces a value between 0 and 1, the next step is to use a rule that allows obtaining 0 or 1. For this, well-defined rules are applied that use the concepts of complement, elite, and random, among others.

#### 3.1.2. Angle Modulation

This method is based on the family of trigonometric functions shown in equation (3). These functions have four parameters responsible for controlling the frequency and displacement of the trigonometric function:

(3) $$\begin{array}{c} g_{i}\left(x_{j}\right)=\text{sin}\left(2\pi \left(x_{j}-a_{i}\right)b_{i}\text{ cos}\left(2\pi \left(x_{j}-a_{i}\right)c_{i}\right)\right)+d_{i}. \end{array}$$

The first time this method was applied to binarizations was in PSO. In this case, binary PSO was applied to benchmark functions. Assume a given binary problem of dimension *n*, and let *X*=(*x*_1_, *x*_2_,…, *x*_*n*_) be a solution. We start with a four-dimensional search space. Each dimension represents a coefficient of equation (3). Then, every solution (*a*_*i*_, *b*_*i*_, *c*_*i*_, *d*_*i*_) is associated with a trigonometric function *g*_*i*_. For each element *x*_*j*_, the following rule is applied:

(4) $$\begin{array}{c} b_{ij}=\left\{\begin{array}{cc} 1, & \text{if }g_{i}\left(x_{j}\right)\geq 0,\\ 0, & \text{otherwise}. \end{array}\right. \end{array}$$

Then, for each initial solution of 4 dimensions (*a*_*i*_, *b*_*i*_, *c*_*i*_, *d*_*i*_), the function *g*_*i*_, which is shown in equation (3), is applied and then equation (4) is utilized. As a result, a binary solution of dimension *n*, (*b*_*i*1_, *b*_*i*2_,…, *b*_in_), is obtained. This is a feasible solution for our *n*-binary problem. The angle modulation method has been applied to network reconfiguration problems [35] using a binary PSO method, to an antenna position problem using an angle modulation binary bat algorithm [36], and to a multiuser detection technique [37] using a binary adaptive evolutionary algorithm.

#### 3.1.3. Quantum Binary Approach

There are three main types of algorithms in research that integrates the areas of evolutionary computation (EC) and quantum computation [38].

1. Quantum evolutionary algorithms: these methods correspond to the design of EC algorithms to be applied in a quantum computing environment
2. Evolutionary-based quantum algorithms: these algorithms attempt to automate the generation of new quantum algorithms using evolutionary algorithms
3. Quantum-inspired evolutionary algorithms: this category uses quantum computing concepts to strengthen EC algorithms

In particular, the quantum binary approach is a type of quantum-inspired evolutionary algorithm. Specifically, this approach adapts the concepts of *q*-bits and superposition used in quantum computing applied to traditional computers.

In the quantum binary approach, each feasible solution has a position *X*=(*x*_1_, *x*_2_,…, *x*_*n*_) and a quantum *q*-bit vector *Q*=[*Q*_1_, *Q*_2_,…, *Q*_*n*_]. *Q* represents the probability of *x*_*j*_ taking the value 1. For each dimension *j*, a random number between [0, 1] is generated and compared with *Q*_*j*_: if rand < *Q*_*j*_, then *x*_*j*_=1; otherwise, *x*_*j*_=0. The upgrade mechanism of the *Q* vector is specific for each metaheuristic.

The main difficulty that general binarization frameworks face is related to the concept of spatial disconnect [39]. A spatial disconnect originates when nearby solutions generated by metaheuristics in the continuous space are not transformed into nearby solutions when applying the binarization process. Roughly speaking, we can think of a loss of the continuity of the framework. The spatial disconnect phenomenon consequently alters the properties of exploration and exploitation, and therefore the precision and convergence of the metaheuristics decrease. A study was conducted on how the TFs affect the exploration and exploitation properties in [40]. For angle modulation, a study was conducted in [39].

On the other hand, specific binarization algorithms that modify the operators of the metaheuristic are susceptible to problems such as Hamming cliffs, loss of precision, search space discretization, and the curse of dimensionality [39]. This was studied by [41] and for the particular case of PSO by [42]. In the latter, the authors observed that the parameters of the Binary PSO change the speed behavior of the original metaheuristic.

### 3.2. Hybridizing Metaheuristics with Machine Learning

Machine learning concerns algorithms that are capable of learning from a dataset [43]. This learning can be supervised, unsupervised, or semisupervised. Usually, these algorithms are used in problems of regression, classification, transformation, dimensionality reduction, time series, anomaly detection, and computational vision [44], among others. On the other hand, metaheuristics correspond to a broad family of algorithms designed to solve complex problems without the need for a deep adaptation of their mechanism when changing problems. They are incomplete techniques and generally have a set of parameters that must be adjusted for proper operation.

When a state-of-the-art integration between metaheuristic and machine learning algorithms is developed, the integration is considered bidirectional. This means that there are studies whereby metaheuristic algorithms contribute to improving the performance of machine learning algorithms, and there are investigations where machine learning algorithms improve the convergence and quality of metaheuristic algorithms. In the case of metaheuristics that improve the performance of machine learning algorithms, we see that integration is found in all areas. In classification problems, we find that such algorithms have been used mainly in feature selection, feature extraction, and the tuning of parameters. In [45], a genetic-SVM algorithm was developed to improve the recognition of breast cancer through image analysis. In this algorithm, genetic algorithms were used for the extraction of characteristics. A multiverse optimizer algorithm was used in [46] to perform the feature selection and parameter tuning of SVM on a robust system architecture. The training of feed-forward neural networks was addressed using an improved monarch butterfly algorithm in [47]. In [48], a geotechnical problem was addressed by integrating a firefly algorithm with the least squares support vector machine technique. For the case of regression problems, we found in [49] an application in the prediction of the compressive strength of high-performance concrete using metaheuristic-optimized least squares support vector regression. The improved prediction of stock prices was addressed in [50], therein integrating metaheuristics and artificial neural networks. Additionally, in [51], a stock price prediction technique was developed using a sliding-window metaheuristic-optimized machine learning regression applied to Taiwan's construction companies. In [52], using a firefly version, the least squares vector regression parameters were optimized with the aim of improving the accuracy of the prediction in engineering design. In the case of unsupervised learning techniques, we find that metaheuristics have contributed significantly to clustering techniques. For example, in [53], an evolutionary-based clustering algorithm that combines a metaheuristic with a kernel intuitionistic fuzzy c-means method was proposed with the aim of designing clustering solutions to apply them to different types of datasets. Also in clustering, centroid search is a problem type that suffers a large algorithmic complexity. This problem consists of the search for centroids with the objective of grouping the set of objects studied in an improved manner. Because this problem is NP-hard, approximation methods have been proposed. For example, an improved artificial bee colony algorithm was developed in [54] with the goal of solving the energy efficiency clustering problem in a wireless sensor network. In [55], a mathematical model and a clustering search metaheuristic were developed for addressing the helicopter transportation planning of oil and gas production platform employees.

On the other hand, when looking for the contributions of machine learning techniques in metaheuristic algorithms, two main lines of research can be distinguished. The first line of research corresponds to specific integrations. In these integrations, machine learning techniques are integrated through a specific operator in one of the modules that establish the metaheuristic. The second line of research explores general integrations, where the machine learning techniques work as a selector of different metaheuristic algorithms, therein choosing the most appropriate for each instance. A metaheuristic, in addition to its evolution mechanism, usually uses solution initiation operators, solution perturbation, population management, binarization, the tuning of parameters, and local search operators, among others. The specific integrations explore the machine learning application on some of these operators. For the case of parameter tuning in [56], the parameter tuning of a chess rating system was implemented. Based on decision trees and using fuzzy logic, a semiautomatic parameter tuning algorithm was designed in [57]. Another relevant area of research is related to the design of binary versions of algorithms that work naturally in continuous spaces. This line of research aims to apply these binary versions in combinatorial problems. In this area, we find in [2] the application of unsupervised learning techniques to perform the binarization process. In [29], the percentile concept was explored in the process of generating binary algorithms. Additionally, in [17], the big data Apache spark framework was applied to manage the size of the solution population to improve the convergence times and quality of results. The randomness mechanism is frequently used for the initialization of the solutions of a metaheuristic. However, machine learning has been used to improve the solution initialization stage. In [58], case-based reasoning was used to initialize a genetic algorithm and apply it to the weighted-circle design problem. Hopfield neural networks were used in [59] to initiate solutions of a genetic algorithm that was used to solve an economic dispatch problem.

When analyzing the methods found in the literature addressing general integrations of machine learning algorithms on metaheuristics, we find three main groups: algorithm selection, hyperheuristics, and cooperative strategies. The objective of algorithm selection is to choose from a set of algorithms and a group of associated characteristics for each instance of the problem an algorithm that performs best for similar instances. In the hyperheuristics strategy, the goal is to automate the design of heuristics or metaheuristic methods to address a wide range of problems. Finally, cooperative strategies consist of combining algorithms in a parallel or sequential manner to obtain more robust methods. The cooperation can be completed by sharing the complete solution or partially when only part of the solution is shared. In [60], the berth scheduling problem at bulk terminals was addressed by algorithm selection techniques. The problem of nurse rostering through a tensor-based hyperheuristic algorithm was addressed in [61]. Finally, a distributed framework based on agents was proposed in [62]. In this case, each agent corresponds to a metaheuristic, and it has the ability to adapt through direct cooperation. This framework was applied to the problem of permutation flow stores.

## 4. Binary Db-Scan Algorithm

**Figure 1:**
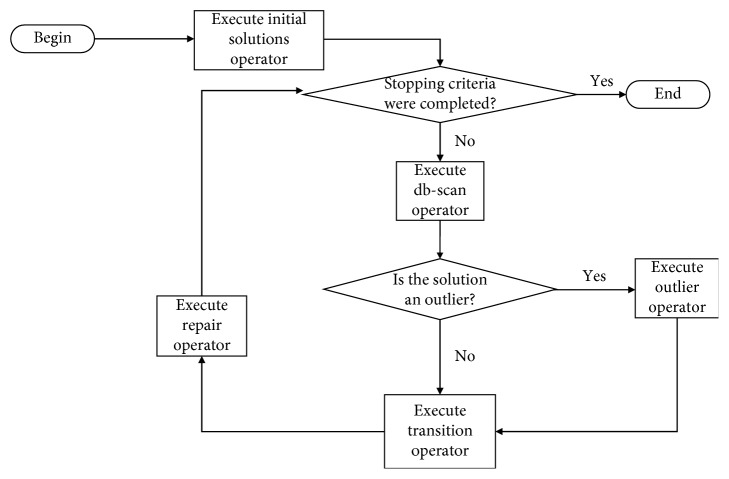
A general flow chart of the binary db-scan algorithm.

The binary db-scan algorithm is composed of five operators. The first operator, which will be detailed in Section 4.1, initializes the solutions. After the solutions are started, the next step is to verify if the maximum iteration criterion is met. When the criterion has not been met, the binary db-scan operator is executed. This operator continuously executes the metaheuristics and then clusters the solutions considering the absolute value of the velocity of the solutions. The details of this operator are described in Section 4.2. Subsequently, using the clusters generated by the db-scan operator, the transition operator will proceed to binarize the solutions generated by the continuous metaheuristics. When points are identified by db-scan as outliers, a transition operator for outliers is applied. The transition operator and the outlier operator are described in Section 4.3. Finally, when the obtained solutions do not satisfy all the restrictions, the repair operator described in Section 4.4 is applied. Additionally, a heuristic operator is used in the initiation and repair of the solutions. This operator is detailed in Section 4.5. The flow diagram of the binary db-scan algorithm is shown in Figure 1.

### 4.1. Initiation Operator

This operator attempts to initiate the solutions that the binary db-scan algorithm will use. The first step, the SelectRandomColumn() function select a column randomly. Then, the operator asks if the row coverage constraint is fulfilled. When the constraint is not met, the initiating operator calls the heuristic operator. This heuristic operator receives the list of columns that currently has the solution and returns a new column to be incorporated. The details of the heuristic operator are described in Section 4.5. The call to the heuristic operator is executed until all rows are covered. The procedure for initiating the solutions is shown in Algorithm 1.

### 4.2. Binary Db-Scan Operator

The goal of the binary db-scan operator is to group the different solutions obtained by the execution of the continuous metaheuristics. When considering solutions as particles, we will understand the position of the particle as the location of the solution in the search space. On the other hand, the velocity represents the transition vector of the particle from iteration *t* to iteration *t*+1.

To perform the clustering, the density-based spatial clustering of applications with noise (db-scan) algorithm is used. Db-scan is a data grouping algorithm proposed in 1996 by [63]. Db-scan uses the concept of density to perform the clustering: given a set of *S* points in a metric space, db-scan groups the points with many nearby neighbors, marking as outliers those that are alone in low-density regions. Db-scan requires two parameters: a radius *ϵ* and a minimum number of neighbors *δ*. The db-scan algorithm can be divided into the following steps:

- Find the points in the *ϵ* neighborhood of every point and identify the core points with more than *δ* neighbors
- Find the connected components of core points on the neighbor graph, ignoring all noncore points
- Assign each noncore point to a nearby cluster if the cluster is an *ϵ* neighbor; otherwise, assign it to noise

Let Mh be a swarm intelligence continuous metaheuristic and List*P*(*t*) be the position list of the solutions given by Mh at iteration *t*. The binary db-scan operator has input parameters Mh and List*P*(*t*) and aims to cluster the solutions given by Mh. The first step of the operator is to iterate the list List*P*(*t*) using Mh to obtain the list of positions List*P*(*t*+1) at iteration *t*+1. Subsequently, using List*P*(*t*) and List*P*(*t*+1), we obtain the list with transition velocities List*V*(*t*+1).

Let *v*^*p*^(*t*+1) ∈ List*V*(*t*+1) be the velocity vector in the transition between *t* and *t*+1 corresponding to particle *p*. This vector has *n* dimensions, where *n* depends on the number of columns that the problem possesses. Let *v*_*i*_^*p*^(*t*+1) ∈ *v*^*p*^(*t*+1) be the value for dimension *i* of the vector *v*^*p*^(*t*+1). Then, List*V*_*i*_(*t*+1) corresponds to the list of absolute values of *v*_*i*_^*p*^(*t*+1), ∀*v*^*p*^(*t*+1) ∈ List*V*(*t*+1). Next, we apply db-scan to the list List*V*_*i*_(*t*+1), thereby obtaining the number of clusters *n*Clusters(*t*+1) and the cluster to which each *v*_*i*_(*t*+1) belongs List*V*_*i*_Clusters(*t*+1), where abs(*v*_*i*_(*t*+1)) ∈ List*V*_*i*_(*t*+1). The procedure for the binary db-scan operator is shown in Algorithm 2.

### 4.3. Transition Operator

The db-scan operator returns the number of clusters and a list with the cluster identifier to which each element belongs: *v*_*i*_^*p*^ ∈ List*V*_*i*_(*t*+1). The purpose of the transition operator is to binarize the solutions generated by Mh and clustered by the binary db-scan operator. To perform the binarization, we must consider that the identifier Id(*J*) ∈ *ℤ* of the clusters will be assigned in the following manner: a value of 0 will be assigned to the cluster that has *v*_*i*_ with the lowest absolute value. Let *v*_*j*_ ∈ *J* and *v*_*i*_ ∈ *I* be elements of clusters *J* and *I*, respectively, and abs(*v*_*j*_) > abs(*v*_*i*_); then, Id(*J*) > Id(*I*). The value of Id will be consecutive integers and if *J* ≠ *I* ⟹ Id(*J*) ≠ Id(*I*). Finally, for the cases identified by db-scan as outliers, we have (Id(Ol)=−1,  where Ol ∈ outliers). Then, each cluster will be assigned a transition probability given by equation (5). In this equation, *α* corresponds to the initial transition coefficient, and *β* corresponds to the transition probability coefficient.

(5) $$\begin{array}{c} P_{tr}\left(J\right)=\alpha +\beta \frac{\text{Id}\left(J\right)}{T}\text{,} \end{array}$$

where *T* is the total number of clusters not considering outliers.

Finally, to execute the binarization process, consider *x*(*t*) as the position of a particle in iteration *t*. Let *x*_*i*_(*t*) be the value of the dimension *i* for the particle *x*(*t*), and let *v*_*i*_^*x*^(*t*+1) be the velocity of the particle *x*(*t*) in the *i* dimension to transform *x*(*t*) from iteration *t* to iteration *t*+1. Additionally, consider that *v*_*i*_^*x*^(*t*+1) ∈ *J*, where *J* is one of the clusters identified by the binary db-scan operator. Then, we use equation (6) to generate the binary position of the particles in iteration *t*+1.

(6) $$\begin{array}{c} x_{i}\left(t+1\right)≔\left\{\begin{array}{cc} {\hat{x}}_{i}\left(t\right), & \text{if rand}<P_{tr}\left(J\right)\text{ where }v_{i}^{x}\left(t+1\right)\in J,\\ x_{i}\left(t\right), & \text{otherwise}. \end{array}\right. \end{array}$$

When *v*_*i*_^*x*^(*t*+1) ∈ outliers, the procedure is as follows: From the complete list of outliers, the *v*_*i*_^*x*^(*t*+1) are ordered, starting with the solution with the best fitness and proceeding to those with the worst performance. The top 20% of solutions in terms of fitness is chosen, and a transition value of *α* is applied. For the remaining elements, a transition value of *α*+*β* is applied. Finally, once the transition operator is applied, a repair operator is used, as described in Section 4.4 for solutions that do not satisfy some of the restrictions. The details of the transition operator are shown in Algorithm 3.

### 4.4. Repair Operator

The repair operator is executed after the execution of the transition operator. In the event that the coverage condition of the rows is not met, the repair operator uses the heuristic operator to add new columns. After all the rows are covered, we verify that there are no groups of columns that cover the same rows. The details of the repair operator are shown in Algorithm 4.

### 4.5. Heuristic Operator

When a solution needs to be started or repaired, a heuristic operator is used that selects a new element. As an input parameter, the operator considers the solution *S*_in_, which needs to be completed. In the case of being a new solution, *S*_in_=∅. With the list of columns belonging to *S*_in_, we obtain the set of rows *R* not covered by the solution. With the set of rows not covered and using equation (7), we obtain in line 4 the best 10 rows to be covered. With this list of rows (listRows) online 5, we obtain the list of the best columns according to the heuristic shown in equation (8). Finally, in line 6, we randomly obtain the column to incorporate. The details of the heuristic operator are shown in Algorithm 5.

(7) $$\begin{array}{c} \text{Weight row}\left(i\right)=\frac{1}{L_{i}}, \end{array}$$

where *L*_*i*_ is the sum of all ones in row *i*.

(8) $$\begin{array}{c} \text{Weight column}\left(j\right)=\frac{c_{j}}{\left|R{\;\cap }^{}M_{j}\right|}, \end{array}$$

where *M*_*j*_ is the set of rows covered by Col*j*.

## 5. Results

To determine the contribution of the db-scan algorithm to the binarization process, three groups of experiments are performed. The first group compares the db-scan algorithm with two random operators, as detailed in Section 5.2. The second group considers comparing db-scan with the *k*-means clustering technique. The results are shown in Section 5.3, and the details of the k-means technique can be found in [1]. Finally, the third group is shown in Section 5.4 and compares the binarization performed by db-scan with the binarization using TFs. The latter is a technique widely used in the binarization of continuous algorithms. Additionally, in Section 5.1, we describe the methodology used to define the parameters of the utilized algorithms.

CS [5] and PSO [7] were the selected algorithms. These algorithms are chosen for three reasons. Both algorithms are quite simple to parameterize; thus, the study can focus on the binarization technique rather than the parameterization. On the other hand, both algorithms have satisfactorily resolved nonlinear optimization problems [17, 32, 64–66]. Finally, simplified theoretical convergence models for both PSO [39] and CS [67] have been developed.

For the evaluation of the db-scan algorithm, instances *E*, *F*, *G*, and *H*, which correspond to the most difficult instances from Beasley's OR library, were used. For the execution of the instances, we used a PC with Windows 10 and an Intel Core i7-8550U processor with 16 GB of RAM. The algorithm was programmed in Python 3.7. To perform the statistical analysis in this study, the nonparametric Wilcoxon signed-rank test and violin charts were used. The analysis was performed by comparing the dispersion, median, and interquartile ranges of the distributions.

### 5.1. Parameter Settings

To obtain the parameters necessary to generate the binary algorithms db-scan-PSO and db-scan-CS, the methodology proposed in [1, 2] was selected. This methodology uses 4 measures defined in equations (9) to (12) to determine the best configuration. To be able to compare the different configurations, there are 4 measures, which are located on a radar chart, and the area under the curve is calculated for each configuration. The configuration with the largest area is selected.

1. The percentage deviation of the best value obtained in the ten executions compared with the best known value (see equation (9))

(9) $$\begin{array}{c} b\text{Solution}=1-\frac{\text{KnownBestValue}-\text{BestValue}}{\text{KnownBestValue}}. \end{array}$$

(2) The percentage deviation of the worst value obtained in the ten executions compared with the best known value (see equation (10))

(10) $$\begin{array}{c} w\text{Solution}=1-\frac{\text{KnownBestValue}-\text{WorstValue}}{\text{KnownBestValue}}. \end{array}$$

(3) The percentage deviation of the average value obtained in the ten executions compared with the best known value (see equation (11))

(11) $$\begin{array}{c} a\text{Solution}=1-\frac{\text{KnownBestValue}-\text{AverageValue}}{\text{KnownBestValue}}. \end{array}$$

(4) The convergence time for the best value in each experiment normalized according to equation (12)

(12) $$\begin{array}{c} n\text{Time}=1-\frac{\text{convergenceTime}-\text{minTime}}{\text{maxTime}-\text{minTime}}. \end{array}$$

For PSO, the coefficients *c*_1_ and *c*_2_ are set to 2. *ω* is linearly decreased from 0.9 to 0.4. For the parameters used by db-scan, the minimum number of neighbors (min*Pts*) is estimated as a percentage of the number of particles (*N*). Specifically, if *N*=50 and min*Pts*=10, then the minimum number of neighbors for the point to be considered within a cluster is 5. To select the parameters, problems *E*.1, *F*.1, *G*.1, and *H*.1 were chosen. The parameter settings are shown in Tables 1 and 2. In both tables, the column labeled Value represents the selected value, and the column labeled Range corresponds to the set of scanned values.

### 5.2. Contribution of Db-Scan Binary Operator

This section attempts to understand the contribution of the db-scan operator when compared with two random operators. The random operator models the situation whereby the transition probability does not depend on the velocity of the particle, unlike the db-scan operator, where the velocity strongly influences the cluster in which it will be located. Two random operators were considered. The first case (naive) is whereby each point has the same probability of transition and therefore is independent of the velocity. In the experiment, two conditions were considered for the random operator. First, the operator *N* random-0.25 has a fixed probability of 0.25; second, the operator *N* random-0.5 uses a fixed transition probability of 0.5. To make the comparison, CS was used. The second random operator, *C* random-5, additionally includes the concept of clusters. In this second operator, 5 clusters are defined, where 5 corresponds to, on average, the clusters obtained by db-scan when executing the different instances. Subsequent to each cluster, a transition probability of the set {0.1, 0.2, 0.3, 0.4, 0.5} is assigned without repetitions. Finally, each particle randomly assigns a cluster.

**Figure 2:**
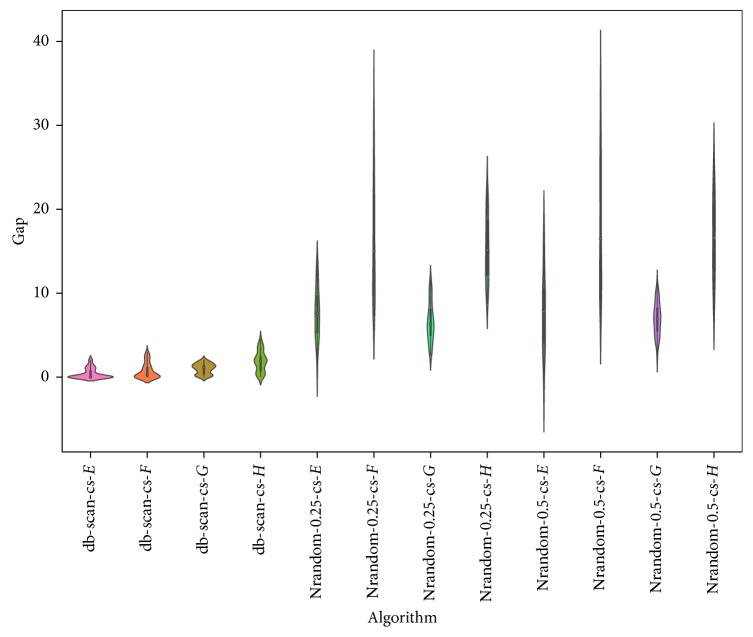
Gap comparison between db-scan and *N*random algorithms for the SCP dataset.

The results obtained using the *N* random operator are shown in Table 3 and Figure 2. When we analyzed the best and average indicators shown in the table, the superiority of the results obtained by db-scan over those obtained by the *N* random-0.5 and *N* random-0.25 operators was observed. This difference is consistent in all instances. The Wilcoxon test indicates that the difference is significant. When analyzing the violin charts, we see that the dispersion, interquartile range, and median are substantially more robust when using the db-scan operator. This experiment is a strong indicator that, in the binarization process, i.e., the assignment of a transition probability to a particle, it is critical to consider the behavior of the particle in the search space. This allows us to obtain better behaving methods.

**Figure 3:**
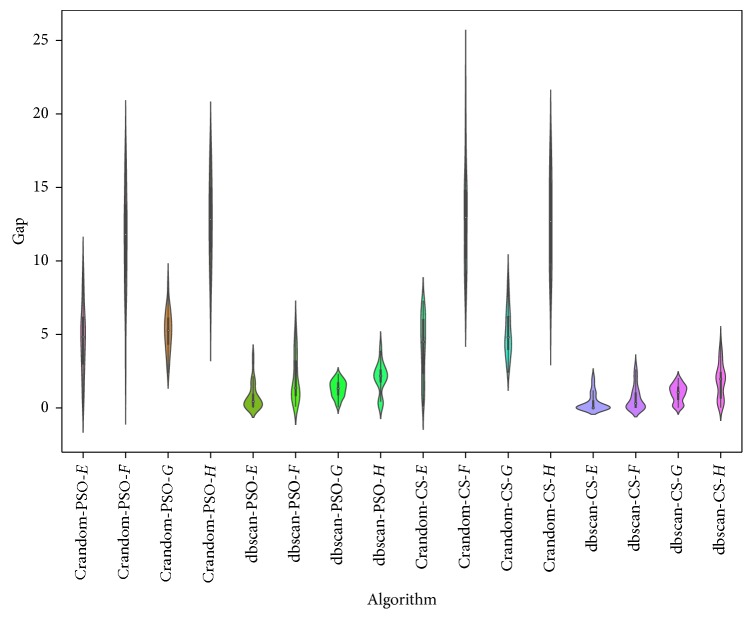
Gap comparison between db-scan and *C*random algorithms for the SCP dataset.

For the *C* random operator, the results are shown in Table 4 and Figure 3. In this experiment, the PSO and CS algorithms were used. When we analyzed the results of the table, it is observed that db-scan has a better behavior than *C* random in both algorithms. When analyzing Figure 3, it is observed that the median, interquartile range, and dispersion measures obtain better results with the db-scan operator. Additionally, we should note that *C* random achieves a better performance than *N* random, which suggests that assigning random transition probabilities by groups is more appropriate than assigning them randomly.

### 5.3. *K*-Means Algorithm Comparison

**Figure 4:**
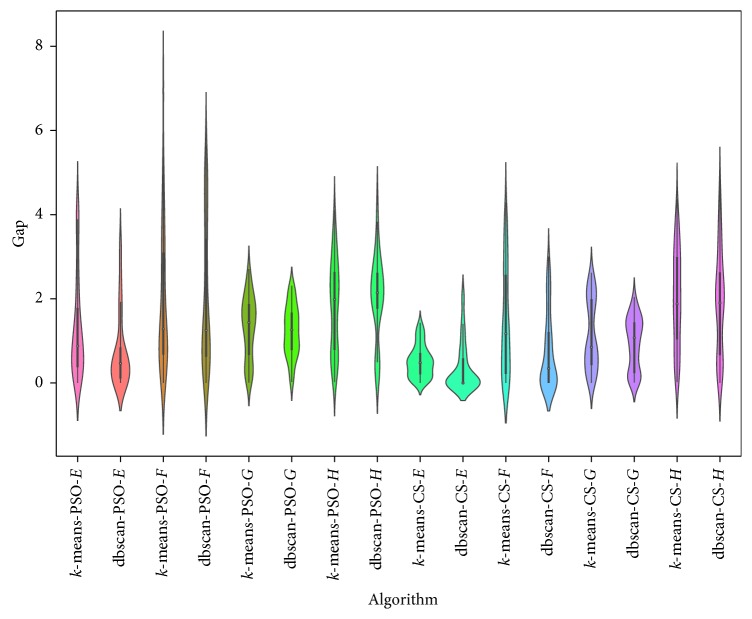
Gap comparison between db-scan and *k*-means algorithms for the SCP dataset.

*K*-means is another clustering technique that was used in [2] to binarize continuous swarm intelligence algorithms and applied to the knapsack problem. The objective of this section is to compare the behavior of the binarization used by db-scan with that used by *k*-means. The *k*-means technique, unlike db-scan, is necessary to define the number of clusters. On the other hand, the computational complexity of *k*-means once the number of clusters (*k*) and the dimension (*d*) of the points are fixed is *O*(*n*^*dk*+1^log *n*), where *n* is the number of points to be clustered. The computational complexity of db-scan is *O*(*n*log*n*). In this experiment, the quality of the solutions and their execution times are compared. For the case of *k*-means, *k* = 5. In the case of db-scan, the number of clusters is variable. For comparison, the same dataset as in the previous experiment is used. In Table 5, the results of the binarization for CS and PSO are shown using the *k*-means and db-scan operators. When we observe the best and average indicators, we see that their values are very similar for both the implementation with *k*-means and the implementation with db-scan. Moreover, when we use the Wilcoxon test, we see that the small differences are not significant. However, when we analyze the execution times, we see that db-scan improves the times obtained by *k*-means. When we compare the interquartile range and the dispersion shown in Figure 4, we see that the results are very similar. Considering that *k*-means handles a fixed number of clusters and given that, in the case of db-scan, this can be variable, the quality of the solutions is not affected significantly.

### 5.4. Transfer Function Comparison

In this section, we detail the experiments that allow us to evaluate the behavior of binarization using db-scan with respect to the TF. The TF is a general binarization mechanism that, instead of using the cluster concept to assign a transition probability, uses functions that map *ℝ*^*n*^ in the space (0,1)^*n*^. Usually, two families of functions are used: v-shape ((*e*^*τ*|*x*_*i*_^*d*^|^ − 1)/(*e*^*τ*|*x*_*i*_^*d*^|^+1)) and s-shape (1/(*e*^−*τx*_*i*_^*d*^^+1)) functions. For more details about TFs, we recommended [32].

**Figure 5:**
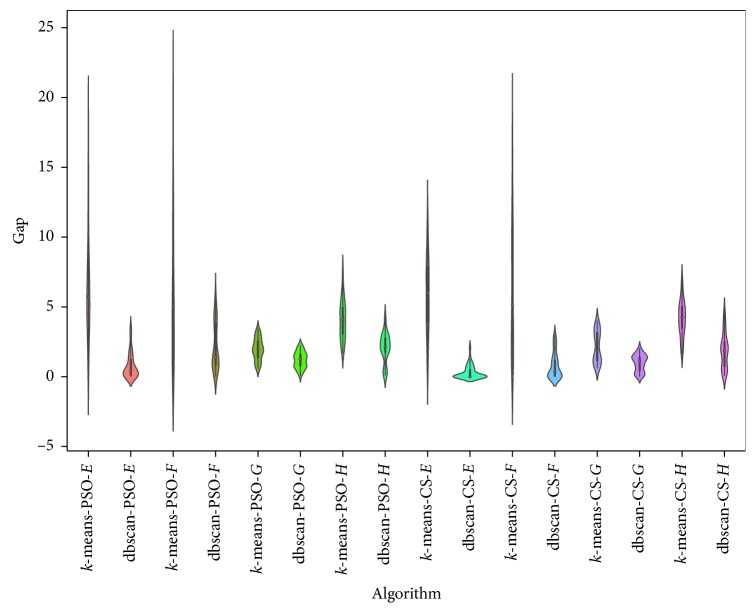
Gap comparison between db-scan and TF algorithms for the SCP dataset.

In our case, we used the v-shape function with the parameter *τ*=2.5 for both the CS and PSO algorithms. The methodology used to determine the family and the parameter *τ* corresponds to the same detailed in Section 5.1. The results are shown in Table 6 and Figure 5. For the TFs, 2000 iterations were considered for the experiment. From Table 6, it is observed when analyzing the best and average indicators that the binary algorithms obtained through db-scan achieve better performance than those obtained with the TF. When performing the Wilcoxon test, the obtained differences are significant. When we look at Figure 5, we see that the dispersion and interquartile ranges are considerably improved when using db-scan. We should note that the TF assigns a particular value of the transition probability to each solution based on a function, unlike db-scan, uses assignment by groups of solutions.

## 6. Real-World Application

The crew scheduling problem (CSP) is related to building the work schedules of crews necessary to cover a planned timetable. The CSP is studied in operations research and is usually related to the airline industry, transit companies, and railways, among others. In this section, we are interested in using the binarizations obtained from applying the db-scan algorithm to the CSP.

The CSP, due to its difficulty, needs to be decomposed in several stages, where each stage has a given computational complexity. The literature contains variations of the CSP. These variations consider integration with other problems or the inclusion of new restrictions. For example, in the CPS in [68], attendance rates were studied. A CSP integration with the vehicle scheduling problem was developed in [69]. In [70], an application of the CSP with fairness preferences was explored. The crew pairing and fleet assignment problems were studied in [71].

The CSP starts with a timetable of services that must be executed with a certain frequency. On the other hand, the service needs to be executed in a certain time window. A service consists of a sequence of trips, where a trip has the following attributes: a start time, an end time, a departure station, an arrival station, and a crew that delivers the service. In terms of the above attributes, each trip is assigned a cost. When we consider a period of time and a crew, a roster must be generated. Then, the CSP consists of finding a subset of rosters that covers all trips at the minimum cost. The problem can be divided into two phases. The first phase corresponds to the generation of a pairing. A pairing is defined as a set of trips that are assigned to a single crew in a short period of time. In this pairing phase, a large number of pairings is generated that satisfy the constraints of the problem. A match must start and end at the same depot, and a cost must be associated. The second phase corresponds to the pairing optimization. At this stage, a selection is made of the best subset of all generated pairings to ensure that all trips are covered at a minimum cost. The modeling of this phase follows an approach based on the solution to set covering or set partitioning problems. In this work, we use a dataset on which the pairs were generated; therefore, we focus our efforts on performing the pairing optimization phase. To verify our algorithm, 7 datasets associated with real-world crew scheduling problems were used. These datasets come from an application from the Italian railways and have been provided by Ceria et al. [72].  Table 7 shows the datasets and their results. When we analyzed the table, we observed that although the problems were larger than the previous problems, the performances of the db*-*scan—PSO and db*-*scan— binarizations were adequate. In the case of db*-*scan—PSO, the gap for the best value was 0.52%, and, on average, it was 1.17%. For db*-*scan—CS, the gap for the best value was 0.52%, and, on average, it was 1.08%.

## 7. Conclusions

In this article, an algorithm was proposed that uses the db-scan technique with the goal of binarizing continuous swarm intelligence metaheuristics. To evaluate the proposed algorithm, as a first step, two random operators were designed with the objective of identifying the contribution of db-scan to the binarization process. Subsequently, the proposed db-scan algorithm was compared with two binarization techniques. The first technique is based on the clustering concept and uses the *k*-means technique, where the number of clusters is fixed. The second technique uses TFs as a binarization mechanism. In the comparison with the binarization technique that uses the concept of i-means, the results were very similar. Those results were confirmed with the Wilcoxon test, which showed no significant differences between the two techniques. However, we must emphasize that the execution times of db-scan were shorter than those of *k*-means. One point to consider is that the different methods of generating the clusters do not affect the quality of the solutions. In the case of k-means, a fixed number of clusters, generated based on the proximity of the points, is defined. For db-scan, the number of clusters is variable and is generated based on the proximity and density of points. In comparison with the TFs, we observed that there is a significant difference in favor of db-scan. This suggests that it is more efficient in the binarization process to assign transition probabilities to groups than to assign them individually. The application of machine learning to metaheuristic algorithms is a line of research that has several aspects. We see that machine learning techniques can learn and help to understand under which conditions a metaheuristic algorithm performs efficiently. However, these techniques can be applied to other operators, such as perturbation operators, when a metaheuristic algorithm is trapped in a local minimum, and operators that control the population of a swarm intelligence algorithm to improve the intensification and diversification properties. Additionally, appealing to the no-free-lunch theorem, it would be interesting to evaluate these algorithms when including machine learning tuning applied to other combinatorial problems.

## Figures and Tables

**Algorithm 1:**
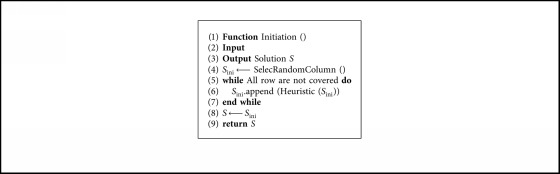
Initialization operator.

**Algorithm 2:**
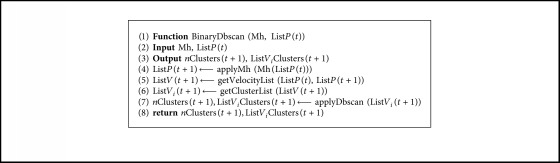
Binary db-scan operator.

**Algorithm 3:**
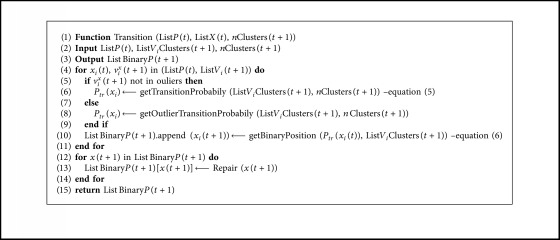
Transition algorithm.

**Algorithm 4:**
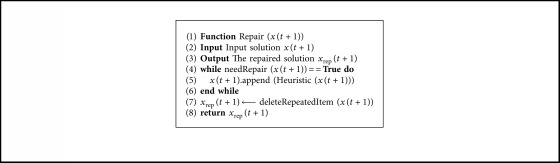
Repair algorithm.

**Algorithm 5:**
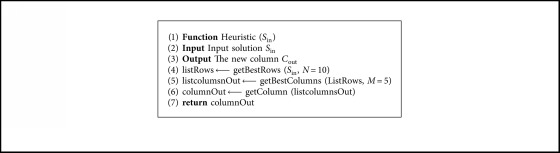
Heuristic operator.

**Table 1:** Parameter setting for PSO Algorithm.

Parameters	Description	Value	Range
*α*	Initial transition coefficient	0.1	[0.08, 0.1, 0.12]
*β*	Transition probability coefficient	0.6	[0.5, 0.6, 0.7]
*N*	Number of particles	50	[30, 40, 50]
*ϵ*	*ϵ* db-scan parameter	0.4	[0.3, 0.4, 0.5]
min*Pts*	Point db-scan parameter	10%	[10, 12, 14]
Iteration number	Maximum iterations	800	[600, 700, 800]

**Table 2:** Parameter setting for CS algorithm.

Parameters	Description	Value	Range
*α*	Transition probability coefficient	0.1	[0.08, 0.1, 0.12]
*β*	Transition probability coefficient	0.5	[0.5, 0.6, 0.7]
*N*	Number of particles	50	[30, 40, 50]
*ϵ*	*ϵ* db-scan parameter	0.4	[0.3, 0.4, 0.5]
min*Pts*	Point db-scan parameter	12%	[10, 12, 14]
*γ*	Step length	0.01	[0.009, 0.01, 0.011]
*κ*	Levy distribution parameter	1.5	[1.4, 1.5, 1.6]
Iteration number	Maximum iterations	800	[600, 700, 800]

**Table 3:** Comparison between db-scan and *N*random operators.

Instance	Best known	db*-*scan-CS			*N*random-0.25-CS			*N*random-0.5-CS		
		Best	Avg	Time (*s*)	Best	Avg	Time (*s*)	Best	Avg	Time (*s*)
*E*.1	29	29	29.0	12.1	29	30.4	7.7	29	30.7	8.2
*E*.2	30	30	30.2	11.8	31	32.6	8.1	30	32.4	7.8
*E*.3	27	27	27.3	12.9	28	29.4	6.6	28	29.8	8.1
*E*.4	28	28	28.0	11.5	29	30.3	6.5	28	30.7	8.3
*E*.5	28	28	28.0	11.4	29	29.8	6.7	28	30.1	8.2

*F*.1	14	14	14.0	12.7	15	16.1	9.1	15	16.9	14.1
*F*.2	15	15	15.2	13.1	16	17.8	8.7	16	18.1	15.3
*F*.3	14	14	14.1	12.6	15	15.4	9.3	15	15.5	14.8
*F*.4	14	14	14.0	12.9	15	16.2	9.4	15	16.2	14.9
*F*.5	13	13	13.2	13.2	14	15.7	8.9	14	15.9	14.1

*G*.1	176	176	177.1	73.1	183	187.4	54.6	184	189.1	60.3
*G*.2	154	156	156.6	72.6	162	167.1	57.3	161	166.3	61.2
*G*.3	166	168	168.4	70.3	174	179.4	58.6	173	178.4	59.7
*G*.4	168	169	169.7	68.9	173	177.2	56.6	174	178.2	60.5
*G*.5	168	168	168.2	72.1	172	176.7	54.1	171	177.8	58.1

*H*.1	63	64	64.8	65.3	68	72.3	52.7	68	73.1	54.9
*H*.2	63	63	63.6	68.1	69	73.1	55.3	68	73.5	53.1
*H*.3	59	60	60.9	69.7	64	68.4	57.2	64	67.9	58.9
*H*.4	58	59	59.2	70.3	63	66.3	56.6	62	67.1	60.4
*H*.5	55	55	55.2	69.3	61	64.2	55.3	60	64.9	59.1

Average	67.1	67.5	67.84	41.2	70.5	73.29	31.97	70.1	73.63	35.0
Wilcoxon *p* − value					1.03*e*−4	8.84*e*−5		5.20*e*−4	8.85*e*−5	

**Table 4:** Comparison between db-scan and *C*random operators.

Instance	Best known	*C*random-5.PSO			db*-*scan-PSO			*C*random-5.CS			db*-*scan-CS		
		Best	Avg	Time	Best	Avg	Time	Best	Avg	Time	Best	Avg	Time (*s*)
*E*.1	29	29	29.9	11.1	29	29.0	13.4	29	29.8	10.6	29	29.0	12.1
*E*.2	30	30	31.1	10.8	30	30.1	13.7	31	31.6	10.9	30	30.2	11.8
*E*.3	27	28	28.7	10.6	27	27.5	14.1	28	28.5	9.8	27	27.3	12.9
*E*.4	28	29	29.9	10.1	28	28.1	12.9	29	29.6	10.2	28	28.0	11.5
*E*.5	28	28	28.7	10.5	28	28.3	13.2	28	28.4	10.4	28	28.0	11.4

*F*.1	14	15	15.5	10.9	14	14.1	12.8	15	15.7	11.3	14	14.0	12.7
*F*.2	15	16	16.8	11.5	15	15.4	13.5	16	16.8	12.1	15	15.2	13.1
*F*.3	14	14	14.9	11.9	14	14.4	13.7	15	15.9	10.9	14	14.1	12.6
*F*.4	14	15	15.8	12.1	14	14.1	13.1	15	15.7	11.2	14	14.0	12.9
*F*.5	13	14	14.7	11.4	13	13.4	13.4	14	15.1	11.4	13	13.2	13.2

*G*.1	176	180	183.9	68.2	176	176.8	81.3	181	184.2	67.2	176	177.1	73.1
*G*.2	154	160	163.8	69.1	156	156.8	77.4	160	164.1	64.3	156	156.6	72.6
*G*.3	166	171	174.6	68.7	168	168.9	79.8	172	175.3	65.1	168	168.4	70.3
*G*.4	168	172	175.1	68.4	169	170.1	78.1	172	174.9	66.3	169	169.7	68.9
*G*.5	168	173	176.4	67.1	169	169.6	81.2	172	175.8	64.8	168	168.2	72.1

*H*.1	63	68	70.6	65.8	64	64.5	74.2	68	70.4	61.4	64	64.8	65.3
*H*.2	63	68	71.2	67.2	64	64.3	73.2	68	71.7	59.7	63	63.6	68.1
*H*.3	59	63	66.1	68.1	60	60.4	72.1	62	65.4	62.3	60	60.9	69.7
*H*.4	58	63	65.9	65.7	59	59.8	76.5	63	66.1	61.8	59	59.2	70.3
*H*.5	55	58	61.5	63.2	55	55.2	74.6	59	62.3	60.2	55	55.2	69.3

Average	67.1	69.7	71.76	39.12	67.6	68.04	45.11	69.85	71.87	37.09	67.5	67.84	41.2
Wilcoxon *p* − value		3.65*e*−4	8.84*e*−5					1.58*e*−4	8.82*e*−5				

**Table 5:** Comparison between db-scan and *k*-means operators.

Instance	Best known	*k*-means.PSO			db*-*scan-PSO			*k*-means.CS			db*-*scan-CS		
		Best	Avg	Time	Best	Avg	Time	Best	Avg	Time	Best	Avg	Time (*s*)
*E*.1	29	29	29.2	17.1	29	29.0	13.4	29	29.1	18.1	29	29.0	12.1
*E*.2	30	30	30.1	18.1	30	30.1	13.7	30	30.2	17.9	30	30.2	11.8
*E*.3	27	27	27.6	16.8	27	27.5	14.1	27	27.1	19.1	27	27.3	12.9
*E*.4	28	28	28.3	17.3	28	28.1	12.9	28	28.2	16.4	28	28.0	11.5
*E*.5	28	28	28.6	17.9	28	28.3	13.2	28	28.2	16.9	28	28.0	11.4

*F*.1	14	14	14.1	17.5	14	14.1	12.8	14	14.1	19.1	14	14.0	12.7
*F*.2	15	15	15.4	18.1	15	15.4	13.5	15	15.3	17.2	15	15.2	13.1
*F*.3	14	14	14.5	18.4	14	14.4	13.7	14	14.2	17.3	14	14.1	12.6
*F*.4	14	14	14.1	17.3	14	14.1	13.1	14	14.3	17.7	14	14.0	12.9
*F*.5	13	13	13.3	17.8	13	13.4	13.4	13	13.0	18.1	13	13.2	13.2

*G*.1	176	176	176.5	98.5	176	176.8	81.3	176	176.8	102.7	176	177.1	73.1
*G*.2	154	156	157.1	95.5	156	156.8	77.4	156	156.9	96.5	156	156.6	72.6
*G*.3	166	168	168.6	93.4	168	168.9	79.8	169	169.7	99.1	168	168.4	70.3
*G*.4	168	169	170.4	103.2	169	170.1	78.1	169	169.4	97.4	169	169.7	68.9
*G*.5	168	168	170.0	101.8	169	169.6	81.2	168	168.4	96.3	168	168.2	72.1

*H*.1	63	64	64.7	99.7	64	64.5	74.2	63	63.6	101.3	64	64.8	65.3
*H*.2	63	63	63.5	101.2	64	64.3	73.2	64	64.5	99.8	63	63.6	68.1
*H*.3	59	60	60.3	96.6	60	60.4	72.1	60	60.8	97.4	60	60.9	69.7
*H*.4	58	59	59.7	97.3	59	59.8	76.5	59	59.7	99.5	59	59.2	70.3
*H*.5	55	55	55.3	98.2	55	55.2	74.6	55	55.4	95.1	55	55.2	69.3

Average	67.1	67.5	68.07	58.09	67.6	68.04	45.11	67.55	67.94	58.14	67.5	67.84	41.2
Wilcoxon *p* − value		0.16	0.42					0.56	0.21				

**Table 6:** Comparison between *db*-scan and *TF* operators.

Instance	Best known	TF-PSO			db*-*scan-PSO			TF-CS			db*-*scan-CS		
		Best	Avg	Time	Best	Avg	Time	Best	Avg	Time	Best	Avg	Time (*s*)
*E*.1	29	29	30.8	47.4	29	29.0	13.4	29	29.7	37.2	29	29.0	12.1
*E*.2	30	30	30.7	41.5	30	30.1	13.7	30	31.3	36.5	30	30.2	11.8
*E*.3	27	28	30.1	39.8	27	27.5	14.1	28	29.2	38.3	27	27.3	12.9
*E*.4	28	29	29.8	45.7	28	28.1	12.9	29	29.7	37.7	28	28.0	11.5
*E*.5	28	29	29.6	44.2	28	28.3	13.2	29	30.1	34.1	28	28.0	11.4

*F*.1	14	14	14.9	46.1	14	14.1	12.8	14	14.9	39.5	14	14.0	12.7
*F*.2	15	15	15.1	49.2	15	15.4	13.5	15	15.2	43.2	15	15.2	13.1
*F*.3	14	14	14.6	49.3	14	14.4	13.7	14	14.9	47.1	14	14.1	12.6
*F*.4	14	14	14.7	45.2	14	14.1	13.1	14	14.8	46.3	14	14.0	12.9
*F*.5	13	14	14.9	41.4	13	13.4	13.4	14	14.7	44.1	13	13.2	13.2

*G*.1	176	177	178.4	286.4	176	176.8	81.3	177	177.9	324.4	176	177.1	73.1
*G*.2	154	157	158.3	301.3	156	156.8	77.4	158	159.1	351.3	156	156.6	72.6
*G*.3	166	169	170.2	314.5	168	168.9	79.8	170	171.4	346.7	168	168.4	70.3
*G*.4	168	169	170.7	322.1	169	170.1	78.1	169	171.2	358.1	169	169.7	68.9
*G*.5	168	169	170.5	303.1	169	169.6	81.2	169	169.9	354.2	168	168.2	72.1

*H*.1	63	64	65.1	265.2	64	64.5	74.2	64	65.1	286.8	64	64.8	65.3
*H*.2	63	64	65.3	246.4	64	64.3	73.2	64	65.7	279.4	63	63.6	68.1
*H*.3	59	60	61.8	298.1	60	60.4	72.1	61	62.1	277.2	60	60.9	69.7
*H*.4	58	59	60.3	293.7	59	59.8	76.5	60	60.6	298.1	59	59.2	70.3
*H*.5	55	56	57.4	300.1	55	55.2	74.6	56	57.2	305.2	55	55.2	69.3

Average	67.1	68.0	69.16	169.03	67.6	68.04	45.11	68.2	69.23	179.27	67.5	67.84	41.2
Wilcoxon *p* − value		4.6*e* − 3	1.2*e* − 4					1.05*e* − 3	1.3*e* − 4				

**Table 7:** Railway crew scheduling problems.

Instance	Row	Col	Density (%)	Best known	db*-*scan-PSO (Best)	db*-*scan-PSO (Avg)	Time (*s*)	db*-*scan-CS (Best)	db*-*scan-CS (Avg)	Time (*s*)
Rail507	507	63009	1.2	174	175	179.4	135.1	174	176.8	127.1
Rail516	516	47311	1.3	182	184	185.9	146.7	183	185.1	151.3
Rail582	582	55515	1.2	211	214	216.3	202.8	214	215.9	198.6
Rail2536	2536	1081841	0.4	690	694	698.1	1225.1	693	698.2	1202.1
Rail2586	2586	920683	0.4	944	948	952.7	1201.5	949	951.2	1301.8
Rail4284	4284	1092610	0.2	1062	1067	1070.9	3154.1	1067	1070.4	3015.3
Rail4872	4872	968672	0.2	1527	1533	1542.8	3700.5	1535	1544.2	3682.1
Average				684.29	687.85	692.3	1395.11	687.86	691.69	1382.61

## Data Availability

The data used to support the findings of this study are available from the corresponding author upon request.
